# Editorial: Boredom: the elephant in the room

**DOI:** 10.3389/fsoc.2024.1443980

**Published:** 2024-07-05

**Authors:** Robert D. Austin, Doris Sommer, Pier Luigi Sacco

**Affiliations:** ^1^Western University, London, ON, Canada; ^2^Harvard University, Cambridge, MA, United States; ^3^University of Chieti-Pescara, Chieti, Italy

**Keywords:** boredom, boredom antecedents, boredom and transgression, domestic violence, intimate partner violence, COVID-19, pandemic (COVID-19), pandemic lockdown

*Boredom is the worst sin because it is the father of other sins…when people are bored, they commit sins*. – Charles Baudelaire, Flowers of Evil

This Research Topic originated from our reading in press reports that cases of domestic violence spiked during the COVID-19 lockdowns (Porter et al., 2021; Uzobo and Ayinmoro, 2023). People who had spent swaths of each day apart, involved in productive activity (work, school), were suddenly forced together in their homes, with little or nothing gainful to do. A result, *pandemic boredom*, was posited as a cause (Sommer et al., 2021), an apparently intolerable stressor that incited violence from apparently mild-mannered (likely mostly male; Walby and Allen, 2004) people. The specifics of how and why were not described in the press, but the consequences were: physical or emotional harm, fear and dread in people (likely mostly female) captive to their domestic situations, places where they had formerly felt safest. The governments that forced this isolation seemed caught off guard, helpless to address these unexpected problems. This Research Topic, then, was conceived to inquire into boredom as a trigger of harmful social effects.

But our focus on the dark side of boredom drew immediate protest. Boredom also, it was pointed out, leads to valued outcomes. It is particularly implicated in creative and artistic expression (Mann and Cadman, 2014; Gomez-Ramirez and Costa, 2017). A description, drawn from our past research interviews, of the beginnings of a renown artist's creative urges, summarizes this positive boredom dynamic:

When I was ten years old…we were visit[ing] my two maiden aunts…going down their street, and although it was a relatively old street, all the houses on it looked the same. And—I can remember this as clear as day—I said to my mother, “When I grow up, I'm going to be an architect, so that people won't have to live in houses that all look alike!”… I thought, “It's so boring. Everybody should have an interesting house, a different one.

Boredom, another artist once told us, “[makes me] look for the surprise, the alteration of the process that is slightly out of my control, thank goodness, and that keeps the whole thing alive.”

Two of our submissions offer theoretical framing of this Research Topic. Sommer draws on philosophies of human development to investigate the link between boredom and violence; importantly, she suggests that violence and aesthetic activity can be considered alternative responses to boredom, and she argues that the latter, obviously preferred response could (should) be actively encouraged. If creative activities are outlets for boredom and therefore deter violence, then providing support for those activities of “symbolic violence” (art) might be granted by governments as a responsibility for harm-prevention.

Levine conceptually maps the idea of boredom, in its negative and positive potentialities. Boredom, it turns out, is not a simple idea. He credits Seneca with first identifying the pattern in which busy life turns to idleness, combines with frustration, mingling self-dislike with feelings of confinement and drooping spirits, to yield to unsteadiness of mind, and jealousy of others' progress. Who or what is responsible (thus what can be done about it) varies with interpretations of the idea. Is boredom a problem in the mindset of the individual that can, given sufficient strength of will, be opted out of? Or does it follow inevitably from the design of human conditions imposed externally (e.g., from alienation from one's labor, as in Adorno, or from one's station in life, as in Virginia Woolf)? Is it moral failing (a choice of idleness when industriousness is viable, thus a sin, according to Baudelaire)? A religious failing (estrangement from God)? A physical disease (humors out of balance)? Then there are positive elements: The urge to flee boredom alerts us to the inadequacy of a current state and provides impetus to something else better. It can be a deserved critique; a thing can deserve the description “boring” and thus petition for variation. It can be revealing of an important truth (e.g., the temporariness of life). Perhaps it even encourages us to do things for the sake of it, rather than to have accomplished it, thus, to live more fully.

Zeißig echoes Sommer's idea that boredom suggests a choice we can make between negative and positive alternatives, in the domain of learning; like Levine, she notes the complexity of the idea, and that different facets of its meaning suggest different impacts on children in school. Tempelaar and Niculescu argue, also, for a multifaceted concept of boredom and derive evidence of the need for such a conception from a detailed empirical study of university students. Other contributors address specific educational contexts. Vuyk et al. study how boredom appears to affect talent development in mathematically capable students in Paraguay. Johnson et al. measure curiosity among Kenyan adolescents. Xu reviews a monograph that examines the impacts of boredom in foreign language classrooms.

Three pieces address the broader societal impacts of boredom. In an opinion piece, Ndetei et al. explore the effects of boredom across African contexts. Miranda-Galarza and Mayer-Foulkes describe responses to the COVID lockdown from people with disabilities in Mexico, documenting negative tendencies toward violence but also more positive outlets in art and activism. Torvisco et al. conduct a textual analysis that mines Spanish newspaper reporting for insights into violence exercised by children against parents.

Two final contributions focus directly on what to do about harmful boredom. Velasco argues that many common claims about boredom and what we know about it are false; we know much more, he says, than is usually claimed, thus the starting point for dealing with the boredom's negative effects is to confess its “myths.” In a final piece, Talbert brings us full circle by casting the negative effects of boredom into the context of criminology, developing a restorative justice approach based on creativity that addresses the antecedents of boredom-incited crime.

The broader subtext of our writing about pandemic boredom is that we humans appear to live in an uneasy relationship with ourselves. Being too much alone with ourselves disaffects us (Alberti, 2019; Hemberg et al., 2019). Pascal attributed “all of the unhappiness of men” to their inability to “stay quietly in their own chamber” (Pensées, 139; though he possibly confounds solitude and boredom—which can surely be achieved in crowds—it is the boredom that arises from solitude that seems most relevant to lockdowns designed to prevent crowds). There is hope, however, in his suggestion that we can learn “how to stay with pleasure at home.” Literature offers positive models. Smilla, the creation of Danish author Peter Hoeg, describes solitude as the “light of grace.” “I never close my door behind me,” she says, “without the awareness that I am carrying out an act of mercy toward myself.”

Together, these varied explorations illustrate both the complexity of the idea of boredom, and provide insights into the significance of it impacts. Perhaps they also offer clues about how we (men especially) might come to know and like ourselves better when are forced to stay alone (or nearly so) in our chambers.

## Author contributions

RA: Conceptualization, Writing – original draft, Writing – review & editing. DS: Writing – original draft, Writing – review & editing. PS: Writing – review & editing.
